# Trends in childhood and adolescent internalizing symptoms: results from Swedish population based twin cohorts

**DOI:** 10.1186/s40359-019-0326-8

**Published:** 2019-08-02

**Authors:** Natalie Durbeej, Karolina Sörman, Eva Norén Selinus, Sebastian Lundström, Paul Lichtenstein, Clara Hellner, Linda Halldner

**Affiliations:** 1 Department of Clinical Neuroscience, Karolinska Institutet, Stockholm, Sweden; 2 Department of Public Health and Caring Sciences, Uppsala University, Uppsala, Sweden; 3 Centre for Clinical Research, County of Västmanland, Uppsala University, Uppsala, Sweden; 4 Institute of Neuroscience and Physiology, Gillberg Neuropsychiatry Centre, Centre of Ethics Law and Mental Health, Gothenburg University, Gothenburg, Sweden; 5 Department of Medical Epidemiology and Biostatistics, Karolinska Institutet, Stockholm, Sweden; 6 Child and Adolescent Psychiatry Research center, BUP Klinisk forskningsenhet, Stockholm, Sweden; 7 Department of Clinical Science, Child and Adolescent Psychiatry, Umeå University, SE-901 87 Umeå, Sweden

**Keywords:** Internalizing, Childhood, Adolescence, Prevalence, Epidemiology

## Abstract

**Background:**

Previous research has noted trends of increasing internalizing problems (e.g., symptoms of depression and anxiety), particularly amongst adolescent girls. Cross-cohort comparisons using identical assessments of both anxiety and depression in youth are lacking, however.

**Methods:**

In this large twin study, we examined trends in internalizing symptoms in samples of 9 year old children and 15 year old adolescents, gathered from successive birth cohorts from 1998 to 2008 (age 9) and 1994–2001 (age 15). Assessments at age 9 were parent-rated, and at age 15 self- and parent-rated. We examined (*i*) the relation between birth cohorts and internalizing symptoms using linear regressions, and (*ii*) whether percentages of participants exceeding scale cut-off scores changed over time, using Cochrane Armitage Trend Tests.

**Results:**

Among 9 year old children, a significantly increasing percentage of participants (both boys and girls) had scores above cut-off on anxiety symptoms, but not on depressive symptoms. At age 15, a significantly increasing percentage of participants (both boys and girls) had scores above cut-off particularly on self-reported internalizing symptoms. On parent-reported internalizing symptoms, only girls demonstrated a corresponding trend.

**Conclusion:**

In line with previous studies, we found small changes over sequential birth cohorts in frequencies of depression and anxiety symptoms in children. Further, these changes were not exclusive to girls.

## Background

Epidemiological studies have indicated that mental health problems in youth are common and tend to persist into adulthood [1]. An estimated 10–20% of children worldwide experience some form of mental health problems [2–4]. Mental health problems in youth are commonly divided into externalizing (e.g., impaired self-regulation, antisocial behavior) and internalizing (e.g., depression, anxiety, hypersensitivity, worry) problems [5]. Internalizing symptoms are associated with a range of difficulties negatively affecting health and everyday life for youths (e.g., impaired self-worth, lack of joy, disrupted appetite and sleep patterns), including increased risk of self-harm and suicide [4]. A relatively common finding is that internalizing symptoms are more prevalent in girls than in boys, particularly during adolescence. Externalizing symptoms, however, tend to be more prevalent in boys than in girls [6, 7]. A meta-analytic review of sex differences in emotion expression involving a large number of participants (*N* = 21 709) from 166 studies in total, demonstrated that girls show more internalizing symptoms than boys overall, even though the differences were small [8]. The term internalizing, broadly referring to symptoms of anxiety, depression and somatic symptoms, has been used for several decades in research [9]. Despite this, a clear-cut definition of the term is still lacking. In the present study, internalizing symptoms specifically refer to symptoms of depression and anxiety assessed with three different scales.

### Trends in mental health symptoms

Understanding time trends in mental health symptoms is one important aspect in the prevention of youth mental illness [1]. During the past decades, there has been an increase in diagnoses and treatment of youth psychiatric disorders [1]. Whether or not this corresponds to increasing levels of internalizing symptoms in community samples is not studied sufficiently so far. Studies investigating temporal trends in psychiatric symptoms have generated conflicting findings [2, 10]. In a systematic review mental health in youth across studies from various cultural contexts (e.g., countries from northern Europe, Australia and North America) was investigated. It encompassed a ten-year period or longer, and demonstrated that temporal fluctuations are dependent on multiple factors including developmental phase, sex and type of symptom [2]. The studies included in this review demonstrated overall increases in internalizing symptoms during the past decade(s) among girls, with more mixed findings for boys [2]. A literature review on surveys conducted in Sweden indicated an increase in mental health problems (e.g., depressive symptoms and worry) among adolescents aged 11–15 between the mid-1980’s and mid-2000’s, with increasing levels particularly among girls [11]. Another Swedish study that included a large sample of adolescents (*N* = 15,000; 15–16 years old) used repeated cross-sectional assessments of psychosomatic health problems during 1988–2005. The results demonstrated successive increases in psychosomatic health problems specifically in girls across the study period [12]. In parallel, a steady increase in mental health care consumption for children aged 13–17 in Stockholm county (where approximately 20% of Swedish children and adolescents reside) has been firmly established since year 2002 [11]. It is unclear whether this reflects an increase in some form of mental health problem or an increased inclination to seek mental health services. Overall, previous research indicates mixed findings on temporal trends in psychiatric symptoms, with findings partly associated with differences in sex and developmental time period. Cross-cohort comparisons can be used to better understand patterns beyond diagnostic changes [1]. There is a lack of studies investigating symptoms of depression and anxiety in representative community pre-adolescent samples using repeated cross-sectional assessments [2, 11].

## Methods

### Study rationale

The rationale for this large population-based study was to examine trends in internalizing symptoms, both anxiety and depression, in consecutive birth cohorts at age 9 (parent-rated) and age 15 (self- and parent-rated). We investigated (*i*) the relation between birth cohorts and internalizing symptoms, and (*ii*) whether the percentage of participants exceeding cut-off scores of the scales changed over time. Investigating temporal trends of internalizing symptoms is an important research endeavor to explore whether the increase in clinical parameters (i.e., diagnoses and treatment) of anxiety and depression during the past decades corresponds to actual increasing levels of internalizing symptoms.

### Participants

Participants were recruited from the Child and Adolescent Twin Study in Sweden (CATSS), which emanates from the Swedish Twin Registry (STR). The CATSS is an ongoing and nation-wide longitudinal study that aims to investigate mental and somatic health in childhood and adolescence, including all twins born in Sweden since July 1992 [13]. When the twins are 9 years old, their parents are invited to participate in a telephone interview that assesses the child’s health status and social environment. At age 15, all twins and their parents are invited to take part in a questionnaire follow-up.

The current study involved samples of children and adolescents 9 and 15 years old, gathered from successive birth cohorts from 1998 to 2008 (age 9) and 1994–2001 (age 15).

The response rate in the total sample at age 9 was 63.5%. The response rates for each birth cohort 1998–2008 respectively were: 69.9, 73.2, 71.4, 69.6, 73.0, 68.1, 60.3, 63.8, 69.2, 59.7, and 44.2%. Furthermore, the response rate in the total sample at age 15 was 60.1%. The response rates for each birth cohort 1994–2001 respectively was: 60.0, 57.4, 72.4, 61.2, 63.1, 54.7, 57.5, and 55.2%. The data collection took place between 2007 and 2017 for children age 9, and between 2009 and 2016 for adolescents age 15.

### Assessment at age 9

The telephone-interview at age 9 included the following parent-rated scales:

The *Screen for Child Anxiety Related Emotional Disorders* (SCARED) is a 41-item screening instrument for anxiety symptoms, based on criteria in the Diagnostic and Statistical Manual of Mental Disorders 4th edition (DSM-IV), in children and adolescents aged 9–18 [14, 15]. Each item is rated on a 3-point Likert scale: from 0 (= *not true*), to 2 (= *true*), with a maximum score of 82. SCARED is one of the most commonly used scales to assess anxiety symptoms in children [16]. It is considered well-suited for the use in community samples [17], across a wide range of cultural contexts [18]. SCARED has demonstrated overall satisfactory psychometric properties (e.g., internal consistency, test-retest reliability) across different types of samples [19, 20], and good discriminant validity (i.e., ability to differentiate children with and without anxious disorders) [19]. Cut-off values for clinical significance range from a total score of 25 (i.e., reflecting suspected anxiety disorder) to 33 [15, 20]. We chose to use the lower cut-off score, given that our sample is a community sample, and also that the scores were low overall. SCARED revealed satisfactory scale reliability (Coefficient *H* = .90 for all participants, .89 for boys and .89 for girls [21].

The *Short Mood and Feelings Questionnaire* (sMFQ) [22] is a 13-item short form of the original 33-item *Mood and Feelings Questionnaire* (MFQ) [23], developed to assess depressive symptoms in children and adolescents 8–18 years old. Each item is rated on a 3-point Likert scale, from 0 (= *no*), to 2 (= *yes*). Psychometric properties for sMFQ are satisfactory, with adequate internal consistency [22, 24–26], validity for parent-rated scores [27, 28], and a strong overlap between sMFQ and the full-length MFQ [25, 26]. Based on a previous study with a similar study population, we chose to use a score of 7 as a cut-off [29]. The sMFQ revealed satisfactory scale reliability (Coefficient *H* = .84 for all participants as well as for boys and for girls [21].

### Assessment at age 15

The assessment at age 15 encompassed self-and parent-rated versions of the *Strength and Difficulties Questionnaire* (SDQ), a 25-item screening measure developed to assess externalizing and internalizing symptoms in children and adolescents 4–16 years old [30]. SDQ consists of five subscales (i.e., conduct problems, hyperactivity, emotional symptoms, peer problems, and prosocial behavior) with five items in each subscale. The current study only included the emotional subscale (i.e., SDQ-Emotional). Each item is rated on a 3-point Likert scale, from 0 (= *not true*), to 2 (= *certainly true*). SDQ is extensively used in different settings (i.e., clinical and community) to screen for internalizing symptoms [31], and it has been incorporated into national surveys of mental health in Swedish children [11]. We chose to use a score of 5 as cut-off, based on official recommendations for a Swedish context [32].

The SDQ-Emotional parent version revealed satisfactory scale reliability for all participants Coefficient *H* = .81 for all participants, .80 for boys and .82 for girls) [21]. Coefficient *H* values for the SDQ-Emotional self-report version were .73 for all participants, .66 for boys and .71 for girls. There was a moderate correlation between the SDQ-Emotional parent version and the SDQ-Emotional self-report version (Pearson’s *r* = .41).

### Statistical analyses

Internalizing symptoms were investigated using descriptive statistics (i.e., means, standard deviations, range, frequencies). Sex differences between completers and non-completers of the scales were investigated through chi-square tests whereas sex differences in scale scores were computed using *t*-test for independent samples. Additionally, Cohen’s *d* was calculated (by dividing the mean difference by the pooled standard deviation) as a measure of effect size. To explore the relation between birth cohorts and internalizing symptoms, linear regressions were calculated. In these analyses, birth cohort served as the independent variable and individual total scale scores (on SCARED, sMFQ, SDQ parent-report and SDQ self-report) served as dependent variables. Prior to the calculations, we checked the distributions of the scores, and considered them approximately normally distributed.

To explore whether the percentage of participants exceeding cut-off scores of the scales changed over time, Cochrane Armitage Trend Tests (two-sided) were computed. Both sets of twins were used in all analyses. A cluster robust sandwich estimator was applied to adjust the standard errors for the nested twin data when computing the regression models. Analyses were computed in the total sample, and with boys and girls separately.

## Results

### Descriptive statistics

The number of participants completing each scale ranged between 9821 and 17562, with an approximately equal distribution of boys and girls (see Table 1 for descriptive statistics of all scales used, for the total group and divided by sex). There were no differences in the proportions of boys and girls between completers and non completers of the SCARED and sMFQ respectively (*χ*^2^ (1, 17816) **=** 3.06, *p* = .080, *χ*^2^ (1, 17816) = 2.01, *p* = .156). However, a larger proportion of boys were found among the completers (48.2%) than among the non-completers (43.2%) of the SDQ-Emotional parent report (*χ*^2^ (1,12643) = 20.94, *p* < .001). In contrast, a larger proportion of girls were found among the completers (54.7%) of the SDQ-Emotional self-report than among the non-completers of this scale (39.4%) (*χ*^2^ (1,12643) = 131.83, *p* < .001). Moreover, among the completers, girls had significantly higher mean scores than boys on all scales except for the sMFQ where boys had slightly higher mean scores than girls. Effect sizes for the sex differences were negligible for the SCARED and sMFQ (Cohen’s *d* = .09 and .04 respectively), small for the SDQ Emotional parent-report (Cohen’s *d* = .38) and large for the SDQ Emotional self-report (Cohen’s *d* = .81).

**Table 1:** Descriptive statistics for all scales used total group and divided by sex

	*M* (*SD*) total	*M* (SD) boys	*M* (SD) girls	*Cohens’d* boys vs girls	*p* boys vs girls	Range (min-max)	*n* (%) boys/girls
Age 9							
SCARED ^a^ *n* = 14979	5.25 (6.57)	4.94 (6.47)	5.56 (6.66)	.09	< .001	0–74	7493 (50.0)/ 7486 (50.0)
sMFQ^a^ *n* = 17562	1.33 (2.96)	1.40 (3.01)	1.27 (2.91)	.04	.004	0–26	8824 (50.2)/ 8738 (49.8)
Age 15^b^							
SDQ-Emotional pr *n* = 9821	1.15 (1.60)	.84 (1.35)	1.44 (1.76)	.38	< .001	0–10	4722 (48.1)/ 5099 (51.9)
SDQ-Emotional sr^c^ *n* = 10821	2.93 (2.28)	2.00 (1.80)	3.70 (2.35)	.81	< .001	0–10	4873 (45.0) 5946 (54.9)

*Note*. *SCARED* Screen for Child Anxiety Related Emotional Disorders, *sMFQ* Short Mood and Feelings Questionnaire, *SDQ* Strength and Difficulties Questionnaire, *sr* self-report, *pr* parent-report

^a^Cohorts 1998–2008

^b^Cohorts 1993–2001

^c^Data on sex missing for two participants

### Parent-rated internalizing symptoms in nine-year old children born 1998–2008

The regression analyses with the SCARED total score as the dependent variable were statistically significant for the total sample, girls and boys respectively (*p* < .001). Additionally, the regression analyses with the sMFQ as the dependent variable were statistically significant for the total sample (*p* = .024) but not for boys and girls separately. The positive beta-coefficients in the regressions reaching significance suggested positive relations between birth cohorts and the SCARED and sMFQ respectively (see Table 2).

**Table 2:** Descriptive statistics for individual birth cohorts and results from linear regressions for SCARED, sMFQ, SDQ parent-report and SDQ self-report

Birth cohorts	Total	Boys	Girls
	*M* (*SD*)	*M* (*SD*)	*M* (*SD*)
SCARED parent-report age 9			
1998	5.2 (6.7), *n* = 865	5.1 (6.9), *n* = 425	5.4 (6.5), *n* = 440
1999	4.7 (5.7), *n* = 1785	4.5 (5.8), *n* = 890	4.9 (5.5), *n* = 895
2000	4.8 (5.8), *n* = 1593	4.3 (5.5), *n* = 786	5.2 (6.1), *n* = 807
2001	4.4 (5.7), *n* = 1579	3.8 (5.1), *n* = 799	4.9 (6.2), *n* = 780
2002	5.0 (6.2), *n* = 1547	4.7 (5.9), *n* = 773	5.4 (6.5), *n* = 774
2003	5.1 (6.8), *n* = 1647	4.6 (6.2), *n* = 841	5.7 (7.4), *n* = 806
2004	5.2 (6.9), *n* = 1189	5.0 (7.0), *n* = 580	5.3 (6.9), *n* = 609
2005	5.5 (6.8), *n* = 1300	5.6 (7.2), *n* = 662	5.5 (6.3), *n* = 638
2006	6.0 (6.9), *n* = 1545	5.9 (7.3), *n* = 784	6.2 (6.6), *n* = 761
2007	6.4 (7.4), *n* = 1310	6.0 (6.9), *n* = 653	6.9 (7.9), *n* = 657
2008	6.6 (8.0), *n* = 619	6.2 (8.5), *n* = 300	6.9 (7.6), *n* = 319
Linear regression	β = .187, *p* < .001 (95% CI: .151–.223)	β = .260, *p* < .001 (95% CI: .145–.245)	β = .260, *p* < .001 (95% CI: .128–.230)
sMFQ parent-report age 9			
1998	1.00 (2.4), *n* = 925	1.0 (2.5), *n* = 458	1.0 (2.4), *n* = 467
1999	1.1 (2.8), *n* = 1981	1.1 (2.7), *n* = 990	1.1 (2.8), *n* = 991
2000	1.1 (2.7), *n* = 1824	1.0 (2.6), *n* = 908	1.1 (2.7), *n* = 916
2001	1.3 (2.9), *n* = 1836	1.3 (2.8), *n* = 938	1.3 (3.0), *n* = 898
2002	1.6 (3.1), *n* = 1903	1.6 (3.2), *n* = 952	1.5 (3.2), *n* = 951
2003	1.5 (3.2), *n* = 1960	1.6 (3.3), *n* = 994	1.4 (3.1), *n* = 966
2004	1.3 (2.7), *n* = 1504	1.4 (2.8), *n* = 751	1.2 (2.6), *n* = 753
2005	1.2 (2.7), *n* = 1596	1.3 (2.8), *n* = 811	1.1 (2.6), *n* = 785
2006	1.5 (3.2), *n* = 1832	1.7 (3.1), *n* = 941	1.4 (3.0), *n* = 891
2007	1.6 (3.3), *n* = 1513	1.7 (3.4), *n* = 748	1.4 (3.2), *n* = 765
2008	1.5 (3.1), *n* = 686	1.6 (3.3), *n* = 332	1.5 (2.9), *n* = 354
Linear regression	β = .005, *p* = .024 (95% CI: .001–.009)	β = .003, *p* = .063 (95% CI: .000–.011)	β = .003, *p* = .184 (95% CI: −.002–.009)
SDQ-Emotional parent-report age 15			
1994	1.1 (1.5), *n* = 1422	.83 (1.4), *n* = 644	1.3 (1.6), *n* = 778
1995	1.1 (1.6), *n* = 1309	.95 (1.5), *n* = 674	1.3 (1.7), *n* = 635
1996	1.1 (1.6), *n* = 1381	.80 (1.3), *n* = 681	1.4 (1.6), *n* = 700
1997	1.2 (1.6), *n* = 1186	.81 (1.3), *n* = 623	1.5 (1.7), *n* = 563
1998	1.2 (1.6), *n* = 1220	.82 (1.4), n = 623	1.4 (1.9), *n* = 575
1999	1.2 (1.6), *n* = 1128	.88 (1.3), *n* = 553	1.5 (1.9), *n* = 575
2000	1.2 (1.7), *n* = 1109	.77 (1.3), *n* = 495	1.6 (1.9), *n* = 571
2001	1.3 (1.7), n = 1109	.81 (1.3), *n* = 503	1.7 (1.89), *n* = 606
Linear regression	β = .220, *p* = .002 (95% CI: −.080--.360)	β = −.009, *p* = .299 (95% CI: −.026–.008)	β = .046, *p* = .001 (95% CI: .025–.066)
SDQ-Emotional self-report age 15			
1994	2.6 (2.2), *n* = 1372	1.7 (1.7), *n* = 578	3.3 (2.3), *n* = 794
1995	2.5 (2.0), *n* = 1251	1.7 (1.8), *n* = 611	2.9 (2.1), *n* = 640
1996	2.9 (2.2), *n* = 1608	2.0 (1.8), *n* = 759	3.6 (2.2), *n* = 849
1997	3.0 (2.2), *n* = 1334	2.1 (1.8), *n* = 636	3.8 (2.3), *n* = 835
1998	3.0 (2.3), *n* = 1443	2.1 (1.8), *n* = 608	3.7 (2.4), n = 835
1999	3.1 (2.4), *n* = 1278	2.1 (1.9), *n* = 583	3.9 (2.4), *n* = 695
2000	3.2 (2.4), *n* = 1266	2.1, (1.8), *n* = 533	4.0 (2.3), *n* = 722
2001	3.3 (2.4), *n* = 1269	2.0 (1.8), *n* = 554	4.3 (2.4), *n* = 713
Linear regression	β = .116, *p* < .001 (95% CI: .097–.135)	β = .054, *p* < .001 (95% CI: .032–.077)	β = .155, *p* < .001 (95% CI: .129–.181)

**Fig. 1:**
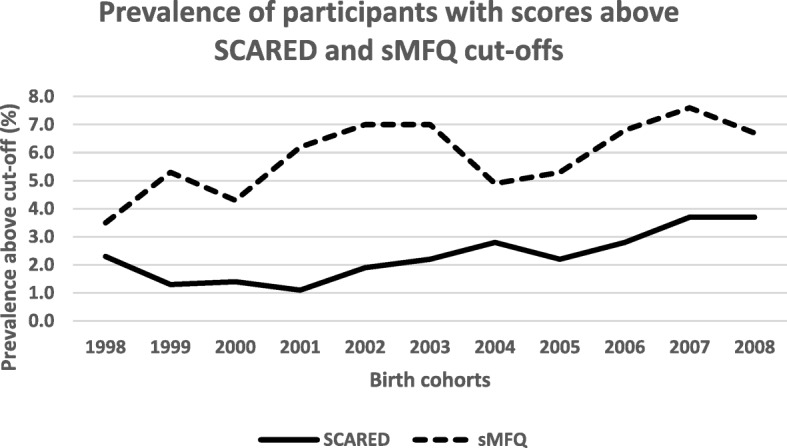
Percentage of participants with scores above cut-offs for the parent-reported Screen for Child Anxiety Related Emotional Disorders (SCARED) (*n* = 324) and the parent-reported Short Mood and Feelings Questionnaire (sMFQ), (*n* = 1044), assessed at age 9

The Cochrane Armitage Trend Test revealed statistically significant trends for a change in the percentage of participants with scores above cut-off on the SCARED for the total sample (*p* < 0.001), and for both boys (*p* < 0.001), and girls (*p* = 0.003) when their results were analyzed separately. There were no such trends for the sMFQ (see Table 3 and Fig. 1).

**Table 3:** Percentage of participants with scores above cut-offs, total sample and individual birth cohorts

Birth cohorts	SCARED parent-report age 9			sMFQ parent-report age 9			SDQ-Emotional parent-report age 15			SDQ-Emotional self-report age 15		
	Total *% n*	Boys *% n*	Girls *% n*	Total *% n*	Boys *% n*	Girls *% n*	Total *% n*	Boys *% n*	Girls *% n*	Total *% n*	Boys *% n*	Girls *% n*
All birth cohorts	2.2 (324)	2.1 (154)	2.3 (170)	5.9 (1044)	6.3 (553)	5.6 (491)	5.0 (495)	2.9 (135)	7.1 (360)	23.6 (2551)	10.0 (489)	34.7 (2061)
1994	n.a	n.a	n.a	n.a	n.a	n.a	4.9 (69)	2.6 (17)	6.7 (52)	19.5 (267)	7.4 (43)	28.2 (224)
1995	n.a	n.a	n.a	n.a	n.a	n.a	5.4 (71)	4.3 (29)	6.6 (42)	15.8 (198)	8.3 (51)	23.0 (147)
1996	n.a	n.a	n.a	n.a	n.a	n.a	3.8 (53)	2.6 (18)	5.0 (35)	23.0 (369)	10.0 (76)	34.5 (293)
1997	n.a	n.a	n.a	n.a	n.a	n.a	4.7 (56)	2.1 (13)	7.6 (43)	23.5 (314)	10.1 (64)	35.8 (250)
1998	2.3 (20)	2.4 (10)	2.3 (10)	3.5 (32)	2.8 (13)	4.1 (19)	5.2 (64)	3.6 (20)	6.6 (44)	24.3 (350)	11.0 (67)	33.9 (283)
1999	1.3 (23)	1.3 (12)	1.2 (11)	5.3 (105)	5.6 (55)	5.0 (50)	5.0 (56)	2.4 (13)	7.5 (43)	26.4 (337)	11.3 (66)	39.0 (271)
2000	1.4 (22)	0.8 (6)	2.0 (16)	4.3 (79)	3.8 (35)	4.8 (44)	5.8 (62)	2.8 (14)	8.4 (48)	26.8 (339)	11.9 (65)	38.0 (274)
2001	1.1 (18)	0.9 (7)	1.4 (11)	6.2 (114)	6.4 (60)	6.0 (54)	5.8 (64)	2.2 (11)	8.7 (53)	29.7 (377)	10.3 (57)	44.7 (319)
2002	1.9 (29)	1.4 (11)	2.3 (18)	7.0 (134)	7.6 (72)	6.5 (62)	n.a	n.a	n.a	n.a	n.a	n.a
2003	2.2 (36)	1.8 (15)	2.6 (21)	7.0 (137)	7.6 (76)	6.3 (61)	n.a	n.a	n.a	n.a	n.a	n.a
2004	2.8 (33)	2.4 (14)	3.1 (19)	4.9 (73)	5.3 (40)	4.4 (33)	n.a	n.a	n.a	n.a	n.a	n.a
2005	2.2 (29)	2.7 (18)	1.7 (11)	5.3 (85)	5.8 (47)	4.8 (38)	n.a	n.a	n.a	n.a	n.a	n.a
2006	2.8 (43)	3.2 (25)	2.4 (18)	6.8 (124)	7.4 (70)	6.1 (54)	n.a	n.a	n.a	n.a	n.a	n.a
2007	3.7 (48)	3.4 (22)	4.0 (26)	7.6 (115)	8.3 (62)	6.9 (53)	n.a	n.a	n.a	n.a	n.a	n.a
2008	3.7 (23)	4.7 (14)	2.8 (9)	6.7 (46)	6.9 (23)	6.5 (23)	n.a	n.a	n.a	n.a	n.a	n.a
Cochrane-Armitage Trend Test Two-sided	*p* < .001	*p* < .001	*p* = .003	*p* = .168	*p* = .218	*p* = .483	*p* = .144	*p* = .261	*p* = .003	*p* = < .001	*p* = .008	*p* = < .001

*Note*. *SCARED* Screen for Child Anxiety Related Emotional Disorders, *sMFQ* Short Mood and Feelings Questionnaire, *SDQ* Strength and Difficulties Questionnaire

^a^Data on sex missing for two participants

### Self-rated internalizing symptoms in 15 year old adolescents, born 1994–2001

**Fig. 2:**
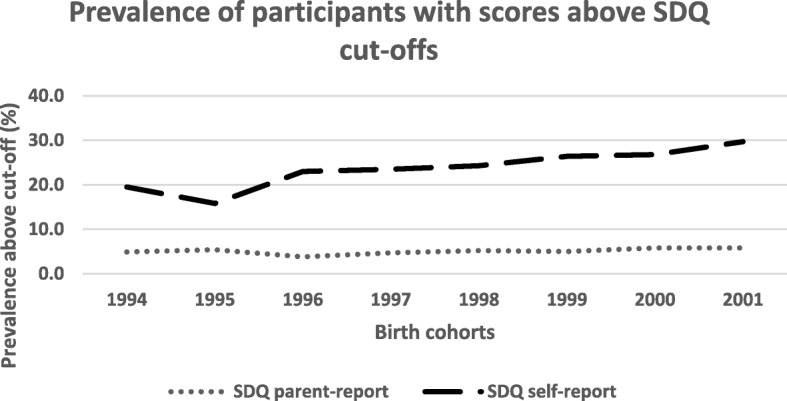
Percentage of participants with scores above cut-offs for the emotional subscale of the Strength and Difficulties Questionnaire (SDQ) parent- (*n* = 495) and self-report (*n* = 2551) versions assessed at age 15

The regression analyses with the SDQ-Emotional *parent-report* version as the dependent variable were statistically significant for the total sample (*p* = .002) and for girls (*p* = .001) but not for boys. The analyses with the SDQ-Emotional *self-report version* 1as dependent variable yielded significant models for the total sample and for both boys and girls (*p* < .001). The positive beta-coefficients in the regressions that reached statistical significance suggested positive relations between birth cohorts and outcomes (see Table 2). The Cochrane Armitage Tests also demonstrated statistically significant trends for a change in the percentage of participants with scores above cut-off on the SDQ-Emotional *self-report version* for the total sample (*p* = < .001), boys (*p* = .008) and girls (*p* < .001) respectively. For girls, there was a corresponding trend of scores above cut-off on the SDQ-Emotional *parent version* (*p* = .003). There were however, no such trends for the total sample, or for boys (see Table 3 and Fig. 2).

## Discussion

This study aimed to examine whether there are time trends in internalizing symptoms, in children and youth. Epidemiological data from high-income countries has demonstrated increases in clinical parameters including diagnosis and treatment of anxiety and depression during the past decades [1]. The current study examines whether such clinical trends correspond to changing levels of internalizing symptoms in population-based samples, using cross-cohort comparisons. The results indicate some trends in levels of internalizing symptoms across birth cohorts. At age 9, the Cochrane Armitage Tests demonstrated an increasing percentage of participants, both boys and girls, with scores above cut-off on anxiety symptoms. There was no such trend for the depressive symptoms. Considering the large sample size of this study, the latter must be reckoned a relatively sound finding. At age 15, there were trends in percentage of participants scoring above cut-off of self-reported internalizing symptoms, for the total sample as well as for boys and girls, respectively. There was a corresponding trend for parent-reported internalizing symptoms for girls but not for boys or for the total sample. Overall, the difference between boys and girls in internalizing symptoms was negligible with regard to scores on the SCARED and sMFQ at age 9. However, there were sex differences with regard to scores on the SDQ parent and self-report scales at age 15 (small and large, respectively).

### Levels of internalizing symptoms

At age 9, levels of parent-rated internalizing symptoms (i.e., symptoms of anxiety and depression assessed with SCARED and sMFQ, respectively; see mean values in Table 1) were very low. Overall, this is to be expected in a non-clinical sample of young children. Comparable levels (including prevalence of participants scoring above cut-off) have been observed in another recent study on a non-clinical sample [33]. It is worth highlighting that our data is based on parent-reports, which could differ significantly from self-ratings by the children (e.g., parents potentially over- or under-estimating the distress of their child).

To our knowledge, there are few studies investigating depression and anxiety symptoms separately for boys and girls [2]. At age 15, girls self-reported substantially higher levels of internalizing symptoms than boys did. Several studies have demonstrated that adolescents have an elevated risk for psychiatric symptoms overall [34]. Specifically, depression and anxiety disorders are typically more common in girls than in boys, especially during adolescent years. This finding is also in line with the Swedish study on levels of psychosomatic health in 15,000 adolescents, demonstrating successive increases in psychosomatic health problems specifically in girls across the study period [12]. Possible explanations for this sex difference might include increased school performance pressure, earlier sexual debut, weight and appearance pressure among girls [2, 12, 34]. The levels of SDQ-scores in the current study seem in line with corresponding figures from previous international studies [35–37]. Even though the levels of internalizing symptoms differed between boys and girls, it is worth noting the increasing percentage of participants with self-reported SDQ-scores above cut-off across time for both boys and girls. These trends could challenge the replicated findings in previous research that increasing levels of anxiety and depression symptoms during the past decades have been relatively specific to girls [1]. Clinicians might be more aware of assessing internalizing symptoms in girls, with the risk of overlooking this set of symptoms in boys.

### Percentages of participants exceeding cut-off scores

At age 9, there was an increasing number of both boys and girls with scores above cut-offs on anxiety symptoms. At age 15, there were increasing numbers of individuals of both sexes exceeding cut-off for self-rated internalizing symptoms. However, such a trend for parent-rated internalizing symptoms was only detected for girls. This trend of increasing percentages exceeding cut-off scores on parent-rated internalizing symptoms was lacking for boys despite the large sample studied. Again, parent-ratings could potentially be underestimating true levels of anxiety and depression symptoms in children. At age 15, the increasing percentage of participants exceeding cut-off is prominent, especially for girls. At the end of the study period, 44.7% of 15-year old girls rated symptoms above cut-off, which is quite remarkable. This result might challenge 5 as a valid cut-off in Sweden when screening for clinical cases with SDQ Emotional in girls at this age. Trends of increasing percentages of participants with scores above cut-offs are relevant from a clinical perspective, since it distinguishes individuals with pronounced symptomatology from participants with milder symptoms.

In contrast to our findings, a meta-analysis of 26 epidemiological studies based on clinical interviews, did not find evidence of increasing rates of child and adolescent depression during a 30-year observation period beginning in the mid 60’s [38]. Also, in a population-based study from Canada, where symptoms of mental illness were assessed bi-annually during the years 1994/1995 to 2008/2009 in large cohorts of Canadian children (*N* > 9000 in each cycle), there were no significant changes in mean scores on depression and anxiety across time for children 10–11 years old and 12–13 years old, but a significant and small increase across time for youth 14–15 years old [10]. Of note, the assessments in the current study were conducted the following decade (between 2007 and 2017 for age 9, and between 2009 and 2016 for age 15). As a clinical reference, there was a certain increase in a main diagnosis of depression among adolescents enrolled in the Stockholm Child and Adolescent Psychiatry (CAP) services: 6.2% of 15-year-old boys were assigned a main diagnosis of depression in 2011, with a corresponding figure of 7.2% in 2016. For girls, the figures were 13.6, and 15.3%, for 2011 and 2016, respectively. Rising levels of internalizing symptoms in youth could indicate an increase of future psychiatric problems, especially if this does not correspond with awareness amongst parents. Increasing percentages of youth scoring above cut-off for internalizing problems may imply psychiatric problems at a level with need for specialized mental health services. This finding might reflect the observed increasing numbers of patients within CAP units.

### Strengths and limitations

There are several unique aspects of the current study. To our knowledge, this is the first population-based study with cross-cohort comparisons, using well-validated scales to assess symptoms of anxiety and depression separately. The CATSS-study is one of the most comprehensive twin studies of childhood mental and somatic health problems ever performed. Given the relatively high response rate (> 60% overall) and the large study cohorts, the sample can be considered representative of the Swedish population. Additionally, this study adds to previous literature by assessing large cohorts and investigating boys and girls separately. Moreover, it includes later cohorts than previously reported in research [2, 11].

The study also had several limitations. Firstly, we did not have separate measures for anxiety and depression at age 15. This is due to sacrifices in terms of extent to prioritize response rate at the follow-ups of the CATSS study [13]. Secondly, there were different raters for participants aged 9 (parent-report) and 15 (self-and parent report). Self-reports of internalizing symptoms could be higher than parent-rated symptoms [33, 39, 42]. Therefore, parent-rated levels of internalizing symptoms at age 9 might be an underestimation of actual symptoms among our participants. However, there is some evidence that parent-information regarding symptoms of anxiety and depression in youth could be as valid as self-rated symptoms (see Table 4 in [40]), and may even be more informative than self-rated information for children specifically (see Table 1 in [41]) In Sweden, mental health problems among youth are low in relation to corresponding figures from other industrialized countries [11]. Therefore, the results are not easily generalized to other cultural contexts. Generalizability from a twin-study might be questioned, even though several studies have suggested that twins are representative of the population at large [43, 44] and that monozygotic and dizygotic twins are similar in personality variation [45]. Previous research on the CATSS population has demonstrated that participants have a higher socioeconomic status compared to non-participants [13]. In population-based surveys, higher levels of psychological problems have been demonstrated in children whose parents have a lower, in contrast to a higher, educational degree [11]. Therefore, the levels of internalizing problems in the current study might not be generalizable to a socioeconomically more diverse population. However, a trend of decreasing response rates would rather underestimate internalizing problems in the present study.

Finally, it is not possible to delineate whether the augmented levels of internalizing symptoms in the current study reflect a real increase in symptoms, an increased tendency to recognize or report these symptoms, or a combination of both. Future longitudinal studies utilizing register-based data linked with self-report should explore whether augmented levels of internalizing symptoms correspond with increasing levels of health care consumption.

To our knowledge, this is the first study using well-validated measures to assess internalizing symptoms, i.e. both anxiety and depressive symptoms, across consecutive birth cohorts of youth. The results demonstrated some trends in internalizing symptoms across birth cohorts, for both boys and girls.

## Conclusions

In the current study including a large Swedish twin sample, slight increases in parent-reported internalizing symptoms at age 9 were observed across the years 2007–2017 for both boys and girls. At age 15, there was an increase in self-reported internalizing symptoms, during the years 2009–2016 for both boys and girls. This study contributes to the field through its methodological strengths; separate assessments of symptoms of depression and anxiety in representative community samples using repeated cross-sectional assessments.

## Data Availability

Datasets are available on reasonable request from the corresponding author.

## Abbreviations

CATSS: Child and Adolescent Twin Study in Sweden
DSM: Diagnostic and Statistical Manual of Mental Disorders
MFQ: Mood and Feelings Questionnaire
SCARED: Screen for Child Anxiety Related Emotional Disorders
SDQ: Strength and Difficulties Questionnaire
sMFQ: Short Mood and Feelings Questionnaire
STR: Swedish Twin Registry
